# Blood–brain barrier and the virus diseases

**DOI:** 10.1590/1980-57642021dn15-030016

**Published:** 2021

**Authors:** Lucas Barbosa Pires Pinheiros, Tales Alexandre Aversi-Ferreira

**Affiliations:** 1 Universidade Federal de Alfenas – Alfenas, MG, Brazil.; 2 Department of Structural Biology, Universidade Federal de Alfenas – Alfenas, MG, Brazil.

Dear Sir,

The blood–brain barrier (BBB) is a specialized structure present in the blood microvasculature of the central nervous system (CNS), which regulates the microenvironment for proper neural functioning; in this way, it protects the CNS from toxins, pathogens, and inflammation.^1^

Historically, the first evidence for the existence of BBB dates to the late 19th century, after Paul Ehrlich injected trypan blue, a direct dye, into the bloodstream of mice. To Ehrlich’s surprise, the dye penetrated almost all tissues of the mice, except the CNS; however, some researchers argued that this was due to the low affinity of the CNS to the dye.

Despite, years later, Ehrlich’s student Edwin Goldmann questioned Ehrlich’s argument after injecting trypan blue directly into the CNS and observing the opposite result, where the CNS changed only its color.^2^ These observations encouraged several studies with different substances injected on the bloodstream and brain’s parenchyma, leading to the concept of BBB;^3^ but in 1967, only the structure of the BBB was first visualized by Reese and Karnovsky with the use of the electron microscopy for biological purposes.^4^

Posterior discoveries, mainly biochemistry ones, show that the BBB is composed of a thin monolayer of non-fenestrated brain endothelial cells (BECs), lining the walls of the CNS blood vessels, maintaining strict contact with pericytes, vascular smooth muscle cells, astrocytes, microglia, and neurons.^2^

This physiological function is performed by the close and specialized connection between the thin layer of brain endothelial cells, connected by tight junctions, and the other cells that compose the BBB, which constitutes the neurovascular unit (NVU).^1^

Accordingly, the loss of integrity of the BBB, via pathological breakdown, was observed in the postmortem brain samples and in the functional imaging of human patients,^5^ for instance, as a result of systemic inflammation that could be an adaptive and appropriate response to systemic upset; however, deleterious effects that are frequently observed are associated with many neurological disorders including multiple sclerosis (MS), stroke, Alzheimer’s disease (AD), epilepsy, traumatic brain injuries, due to the alteration of BBB’s elements such as tight junctions, transporters, denudation of glycocalyx, endothelial cell damage, transcytosis, and LAM expression.

In addition, systemic inflammation changes the permeability of the BBB; in this way, recent data indicate a brain infection caused by the severe acute respiratory syndrome coronavirus 2 (SARS-CoV-2). SARS-CoV-2 is a novel beta coronavirus which causes a highly contagious respiratory problem called coronavirus disease 2019 (COVID-19).

It is a logical thinking that if the virus that causes the brain infection might have crossed the BBB, then the protective properties of the BBB fail. Indeed, a few, but increasing, cases of patients presenting COVID-19 have manifested neurological complications indicating that SARS-CoV-2 can cross the BBB.

As SARS-CoV-2 virus presents the capability to pass through the epithelium like the respiratory and gastrointestinal viruses, it is expected to pass through the BBB and hence infect the peripheral nerves.^6^

Some possible routes could be used by the virus (for a more detailed review, see Alam et al.^1^). Other no less important situation is the long-term neurological complications such as neurodegenerative diseases.

These recent data indicate the imperious need of studies to seek drugs to prevent the SARS-CoV-2 across the BBB and a preparation of health professionals to accompany the patients post-COVID-19 infection and provide fast treatment to prevent the complications of neural morbidities.

Medical monitoring must be accomplished post SARS-CoV-2 infection and front of any neural complication a psychiatric and neurologist must be indicating as a prevention.

It is necessary to call attention for the need of medicines to prevent BBB virus infections in general and the SARS-CoV-2 infection in particular (because it is highly contagious), added to medical attention with infected patients and with patients post-infection.
